# Perspectives of frontline health workers on Ghana’s National Health Insurance Scheme before and after community engagement interventions

**DOI:** 10.1186/s12913-016-1438-y

**Published:** 2016-05-28

**Authors:** Robert Kaba Alhassan, Edward Nketiah-Amponsah, Nicole Spieker, Daniel Kojo Arhinful, Tobias F. Rinke de Wit

**Affiliations:** Department of Economics, University of Ghana, Legon, Ghana; PharmAccess Foundation, Amsterdam, Netherlands; Department of Epidemiology, Noguchi Memorial Institute for Medical Research, University of Ghana, Legon, Ghana; Amsterdam Institute for Global Health and Development, University of Amsterdam, Amsterdam, Netherlands

**Keywords:** Systematic community engagement interventions, National Health Insurance Scheme, Quality service, Frontline health workers, Perspectives, Ghana

## Abstract

**Background:**

Barely a decade after introduction of Ghana’s National Health Insurance Scheme (NHIS), significant successes have been recorded in universal access to basic healthcare services. However, sustainability of the scheme is increasingly threatened by concerns on quality of health service delivery in NHIS-accredited health facilities coupled with stakeholders’ discontentment with the operational and administrative challenges confronting the NHIS. The study sought to ascertain whether or not Systematic Community Engagement (SCE) interventions have a significant effect on frontline health workers’ perspectives on the NHIS and its impact on quality health service delivery.

**Methods:**

The study is a randomized cluster trial involving clinical and non-clinical frontline health workers (*n* = 234) interviewed at baseline and follow-up in the Greater Accra and Western regions of Ghana. Individual respondents were chosen from within each intervention and control groupings. Difference-in-difference estimations and propensity score matching were performed to determine impact of SCE on staff perceptions of the NHIS. The main outcome measure of interest was staff perception of the NHIS based on eight (8) factor-analyzed quality service parameters.

**Results:**

Staff interviewed in intervention facilities appeared to perceive the NHIS more positively in terms of its impact on “availability and quality of drugs (*p* < 0.05)” and “workload on health staff/infrastructure” than those interviewed in control facilities (*p* < 0.1). Delayed reimbursement of service providers remained a key concern to over 70 % of respondents in control and intervention health facilities.

**Conclusion:**

Community engagement in quality service assessment is a potential useful strategy towards empowering communities while promoting frontline health workers’ interest, goodwill and active participation in Ghana’s NHIS.

## Background

Sustainable healthcare financing and universal access to healthcare are important health system goals of many countries globally. To promote attainment of these goals, many Lower and Middle Income Countries (LMICs) have resorted to Social Health Insurance (SHI) to finance healthcare. Ghana’s National Health Insurance Scheme (NHIS) is one such SHI policy introduced in 2003 (Act 650, 2003 amended Act 852, 2012).

The NHIS is currently operational in over 150 district offices nationwide with an active membership of 8.8 million, representing about 35 % of the Ghanaian population, and over 90 % of the disease burden of Ghanaians covered including medical emergencies and accidents [1]. Out of the 3,575 health facilities accredited by the National Health Insurance Authority (NHIA) as at 2012, 53.6 % were owned by government; 39.8 % by private-for-profit; 5.8 % by mission/faith-based and 0.8 % by quasi-government facilities [1].

Since its introduction, the NHIS has contributed to improved health service utilization and health outcomes [2–7]. Outpatient visits per capita improved from 0.37 in 1997 to 1.16 in 2013; likewise, percentage of skilled deliveries improved from 44.5 % in 2006 to 55.0 % in 2013 [8].

Notwithstanding these positive contributions, there are emerging concerns on the quality of health service delivery in NHIS-accredited health facilities [9–14].

Some literature suggest that introduction of the NHIS has contributed to increased doctor-patient ratios in the Northern region of Ghana from 1:8,805 in 2006 to 1:21,751 in 2011 while the nurse-patient ratio worsened from 1:279 in 2006 to 1:1,547 in 2011 [12]. It is also argued the NHIS has compounded challenges in client waiting times, quality of time spent per patient and staff attitude towards clients [14, 15]. Delayed reimbursement of service providers, delayed production and issuance of membership cards and administrative lapses in NHIS district offices also remain substantial concerns to stakeholders of the NHIS in recent times [12, 15].

Even though these challenges have been explored from clients’ perspectives in previous studies [3, 5], not much is known of frontline health workers’ views on the NHIS. Moreover, not many known randomized cluster trials and related studies have been conducted on how community engagement interventions potentially influence frontline health workers’ experiences of the NHIS in Ghana although community participation in health service planning and implementation is a key principle in the Alma-Ata Declaration of 1978 [16].

Previous studies have demonstrated the relevance of community engagement in effective and sustainable implementation of health policies in Ghana [17–20] and other countries [21–26]. Client/community participation in quality service assessment in healthcare and health insurance facilities is conventionally limited to client satisfaction surveys albeit this strategy is increasingly proving ineffective because of potential biased assessment emanating from client intimidation [27]. Limitations of these conventional approaches underscore the need to complement existing strategies with structured community engagement interventions.

Morgan and Lifshay [28] cited in Alhassan et al. [29] defined community engagement in the context of public health as dynamic relationships and dialogue between community members and local health professionals with varying degrees of community and higher level health authorities’ involvement in decision-making and control. Community engagement is critical for sustainable and equitable health policies because community members best define and prioritize their health needs [23].

In this study, Systematic Community Engagement (SCE) interventions were designed and implemented for nearly one year to engage existing community groups and associations in the monitoring and assessment of quality service delivery in NHIS-accredited health facilities and NHIS district offices. For the purposes of this paper, results of the interventions are not discussed instead aggregate effect of the interventions on staff perceptions and experiences of the NHIS and its effects of quality health service delivery are evaluated. The hypothesis is that in health facilities and NHIS district offices where SCE is implemented, frontline health workers’ experiences and perspectives on the NHIS will be enhanced than staff in control facilities.

## Methods

### Study design, population and setting

The study is a randomized cluster trial conducted in 64 NHIS-accredited clinics/health centres and 16 NHIS district offices in the Greater and Western regions of Ghana. Individual respondents were chosen from within each intervention and control groupings. The 64 facilities constitute about 5 % of the 1,180 accredited clinics/health centres in Ghana [1]. Clinical and non-clinical frontline health workers with at least 6 months work experience were eligible to participate in the baseline and post intervention surveys. Randomized cluster trial was deemed appropriate because it is one of the most scientifically rigorous methods of hypothesis testing [30] and the gold standard trial for evaluating effectiveness of interventions while preventing selection bias [31].

### Design and implementation of SCE Interventions

Two categories of SCE interventions were implemented namely: *MyCare* (also called Intensive Engagement) and Light Engagement (LE). The LE intervention used existing community groups/associations to identify service delivery gaps in healthcare facilities and NHIS district offices. The *MyCare* component engaged clients and relevant stakeholders in a participatory process; the focus was on individual clients contrary to the group approach in the LE. Both categories of interventions were implemented and evaluated concurrently from June, 2013 to March, 2014. This paper emphasizes the LE arm of the SCE interventions because the MyCare intervention was implemented in only 6 out of the 32 intervention facilities.

**Fig. 1:**
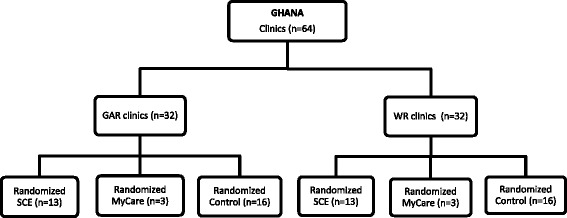
Interventions design and randomization. Source: WOTRO-COHEiSION Ghana Project baseline and follow-up field data (2014); Legend: GAR: Greater Accra Region; WR: Western Region; SCE: Systematic Community Engagement; n=sample size. NOTE: MyCare intervention is not the focus of this paper thus it is not elaborated in subsequent sections of the paper

The SCE interventions were implemented in 32 randomly selected clinics/health centres and their catchment area; of the 32 health facilities that were randomly picked to receive SCE, 26 were randomly assigned the LE intervention and 6 assigned the *MyCare* intervention. The remaining 32 health facilities were used as controls. In each region, NHIS district offices serving the intervention clinics/health centres were also assigned as proxy intervention NHIS district offices. Figure 1 illustrates the randomization into the control, LE and MyCare arms of the study. Moreover, detailed description of the LE and MyCare components of the SCEIs are presented in Table 1.

**Table 1:** Description of the Systematic Community Engagement (SCE) Interventions

**Description of the Systematic Community Engagement (SCE) interventions** **Light Engagement (LE) Intervention** The LE intervention comprised of five steps that actively engaged clients in their communities to rate service quality in their nearest health facility and NHIS district office using predefined quality service proxies. The five implementation steps are: **Step 1:** Recruitment and training of 52 facilitators, and identification of existing community groups/associations. One facilitator was assigned to each of the of 52 community groups in the two study regions (26 in each region). Eligibility criteria for selection of community groups included: documented evidence of routine meetings (at least four times a year); regular meeting venue; clear leadership structure; non-partisan, and active membership not less than an intuitive number of ten (10). The community groups comprised of 22 religious/faith-based groups; 8 traders groups; 1 widows group; 3 community volunteers groups; 3 musician groups; 5 artisans groups and 11 youth groups. Average group size was 29 members (SD = 20). **Step 2:** First round of assessment of service quality based on group members’ most recent (at most 6 months) experiences with the intervention service providers. Service quality indicators at healthcare provider level were: attitude of staff; punctuality of staff; availability of drugs; information provision; opportunity for feedback. Indicators for the health insurer are: information provision; (re)enrolment; delivering what is promised, and opportunity for feedback. A proxy indicator called Net Promotor Score (NPS) was used to measure clients’ trust for service providers. During the assessment, community members were asked to rank their experiences of service quality on a Five point Likert scale ranging from 1 “Very disappointing” to 5 “Very satisfactory”, using a community score card. These same service quality indicators were used for the *MyCare* arm of the SCE interventions. **Step 3:** Regional level validation and feedback sessions to disseminate the group assessment findings with facility heads, clients and NHIA representatives. This platform provided the service providers the opportunity to recognize and accept gaps in healthcare quality and agree on quality improvement plans with timelines and responsible persons. **Step 4:** Follow-up on the service providers by facilitators (3 months after validation and feedback sessions) to ensure implementation of agreed action plans towards quality improvement. **Step 5:** Rewarding best performing health facilities after a second round of community assessment (approximately six months after the first assessment). A citation plaque of honor and a token financial incentive of about US$ 280.0 was awarded best performing facilities to encourage competition among peers towards quality improvement. **Intensive Engagement (** *MyCare* **) Intervention** This component of the SCE interventions was implemented within the catchment area of intervention health facilities using a cyclical process involving clients, health care providers, and the NHIS district offices. Implementation of the *MyCare* arm of the SCE interventions involved six (6) cyclical steps namely: **Step 1:** Recruitment and training of facilitators for the intervention activities. **Step 2:** Semi-quantitative process where 30–50 clients (with evidence of NHIS active membership) were recruited at the exit of the intervention health facilities and later interviewed at home. Assessment of service quality focused on 10 predefined indicators related to service quality at the levels of the healthcare provider and health insurer (see LE interventions described earlier). **Step 3:** Qualitative validation and feedback on semi-quantitative data with community representatives. Six (6) focused group discussions (1 in each catchment area) were conducted to discuss findings of the semi-quantitative interviews and action points taken to address identified service quality gaps. **Step 4:** Briefing: intervention clinics and NHIS district offices were briefed on clients’ experiences of service quality. **Step 5:** Joint stakeholder meeting where representatives of clients/community, healthcare providers, health insurers and regional/district level policy makers were invited to discuss and address identified gaps; a liaison person at the community follows up on the service providers to ensure action plans towards quality service improvement are implemented. **Step 6:** Progress qualitative phase where clients are followed-up six (6) months after the joint stakeholder meeting to determine perceived changes in service quality. Service providers perceived to have improved were rewarded with financial incentives and a citation plague of appreciation.	

### Sampling procedures

The Greater Accra and Western regions were purposively sampled for rural–urban balance since the former is predominantly urban and the latter largely rural; 333 questionnaires were randomly administered to frontline health workers in all 64 sampled health facilities during the baseline survey in 2012. Out of this number, 324 questionnaires were correctly filled and returned representing 97 % return rate; 320 questionnaires were administered during follow-up in 2014 and out of this number, 308 were correctly filled and returned, representing 96 % return rate. Out of the 308 follow-up respondents, 234 were initially interviewed at baseline. Drop-out staff could not be followed because of transfers, deaths, resignations and retirements.

### Data collection

Structured questionnaires consisting of open and close ended questions were used for the data collection before and after the SCE interventions. Questionnaires were administered by trained research assistants for nearly one month in the two study regions. During the interviews, respondents were asked questions related to their age, gender, education, professional category, work experience, religion, monthly salary, and health insurance status. Respondents were also asked to rank their perceptions on the NHIS based on pre-defined indicators on a Likert scale of 1 = “very disappointing” to “4 = “very excellent”. Questions were also asked on perceived impact of the NHIS on quality service delivery based on a scale of 1 = “very large extent” to 4 = “very little extent”.

To ensure effect of the interventions were appropriately evaluated, staff perception questions were similar to the indicators used for the SCE. Thus, quality service markers such as staff attitudes, information provision, client waiting times, NHIS enrolment and renewal process and feedback systems were rated by health staff before and after the interventions.

Piloting of data collection instruments was done in two health facilities in the Greater Accra region and were found to be acceptable and feasible for full-scale implementation.

### Statistical analysis

All data sets were analyzed with the STATA statistical software (version 12.0) after data cleaning and coding to anonymize the responses. Only responses of health workers (*n* = 234) successfully interviewed during baseline and follow-up surveys were maintained for the final analysis (i.e. 468 pooled responses); all analysis were done on “intention to treat” basis.

Socio-demographics and work characteristics of respondents were estimated using proportions; Pearson Chi-square test and paired *t*-test were used as appropriate to determine the differences in these socio-demographic characteristics among staff interviewed in intervention and control groups.

The main outcome variables of interest in the analysis were staff perceptions on the NHIS and its impact on quality service delivery. These outcome variables were derived from 28 Likert scale questions (i.e. 19 questions on staff experiences with NHIS and 9 questions on perceived impact of NHIS on quality health service delivery); the average scale reliability coefficient was tested and found to be 0.77, above the 0.70 rule of thumb [32]. Reverse coding was done for the Likert scale ranging from 1 = “very large extent” to 4 = “very little extent” because some questions were posed in the negative [32]. Higher summated scores thus depict positive perceptions of staff and *vice versa*. Coding for the Likert scale of 1 = “very disappointing” to “4 = “very excellent” was maintained.

The 28 Likert scale questions were factor-analyzed into 8 perception variables using principal component factor analysis with varimax horst rotation (Kaiser off). The perception variables were predicted and named as follows: factor1 = “Feedback channels and stakeholder engagement”; factor2 = “Information provision, adequacy and accessibility”; factor3 = “Availability and quality of drugs covered by NHIS”; factor4 = “Reimbursements and benefits package”; factor5 = “Trustworthiness and complaint handling”; factor6 = “Workload and impact on health resources”; factor7 = “Client waiting time and queuing system”; and factor8 = “Quality of time spent per client”.

To determine effect of the SCEIs on respondents’ perceptions, difference-in-difference (DiD) estimations and propensity score matching (nearest neighbor algorithm) were performed using the pooled baseline and follow-up data (*n* = 468). DiD test was performed to ascertain the mean perception differences among staff in intervention and control facilities using the pooled baseline and follow-up datasets [33].

Even though the study design is a randomized cluster trial, the psmatch2 was employed to determine the treatment effect of the SCE on staff perception variables [34]. This approach was deemed necessary because the staff were not randomly assigned to treatment and control facilities which has the potential to introduce selection bias [35].

Randomization into control and intervention groups was done at the health facility level, not the staff level. Hence, staff who by chance were found in intervention or control clinics were interviewed at random. Potential effect of covariates such as staff gender, age, education, professional category, monthly salary, insurance status, marital status, religious affiliation, clinic location (rural/urban), and clinic ownership (private/public) were accounted for in the estimations.

## Results

### Profile of health workers

Out of the 324 staff interviewed at baseline, 234 (72 %) were successfully interviewed at follow-up. The pooled baseline and follow-up responses were therefore 468 comprising 253 (54 %) from the control group and 215 (46 %) from intervention group. The proportion of females dominated among the 468 respondents in the intervention (60.5 %) and control groups (69.6 %) (*p* < 0.05). Likewise, the proportion of staff who had at least tertiary education was higher in intervention (72.1 %) and control (79.4 %) groups (*p* < 0.1).

Majority of the staff interviewed in intervention (61 %) and control (70 %) health facilities belonged a professional association (*p* < 0.1). A significant proportion of the staff interviewed in intervention facilities also worked in privately owned health facilities while majority of the staff interviewed in control health facilities also worked in public/government owned facilities (*p* < 0.05). There were no statistically significant differences between intervention and control facilities in terms of staff professional category; marital status; religious affiliation; monthly salary and health insurance status (see Table 2).

**Table 2:** Profile of health workers interviewed in intervention and control facilities

		Intervention	Control	
Staff characteristics	^b^Obs.	^a^Mean (Std. Dev.)	^a^Mean (Std. Dev.)	*p*-value
Age	213	37.4(13.1)	37.6(13.1)	0.9129
Gender	Obs.	Proportion (95 % CI)	Proportion (95 % CI)	
Males	468	39.5 (33.0 46.1)	30.4 (24.7 36.1)	0.039**
Females		60.5 (53.9 67.0)	69.6 (63.9 75.3)	
Education				
Below tertiary	468	27.9 (21.9 33.9)	20.6 (15.6 25.6)	0.063*
Tertiary		72.1 (66.1 78.1)	79.4 (74.4 84.4)	
Professional category				
Clinical staff^c^	468	74.4 (68.6 80.3)	76.3 (71.0 81.5)	0.640
Non-clinical staff^d^		25.6 (19.7 31.4)	23.7 (18.5 29.0)	
Marital status				
Married	465	43.9 (37.2 50.6)	45.0 (38.8 51.2)	0.813
Not married		56.1 (49.4 62.8)	55.0 (48.8 61.2)	
Religion				
Christian	466	96.7 (94.3 99.1)	95.6 (93.1 98.2)	0.541
Other		3.3 (0.9 5.7)	4.4 (1.8 6.9)	
Monthly salary				
>GHC 1,300	458	7.1 (3.6 10.5)	10.2 (6.4 14.0)	0.243
<GHC 1,300		92.9 (89.5 96.4)	89.8 (86.0 93.6)	
Belong to professional association				
Yes	320	61.0 (52.9 69.1)	70.4 (63.7 77.1)	0.078*
No		39.0 (30.9 47.1)	29.6 (22.9 36.3)	
Region of work				
Greater Accra	468	51.2 (44.4 57.9)	51.8 (45.6 58.0)	0.894
Western		48.8 (42.1 55.6)	48.2 (42.0 54.4)	
Clinic ownership				
Private	468	59.1 (52.5 65.7)	47.4 (41.2 53.6)	0.012**
Public		40.9 (34.3 47.5)	52.6 (46.4 58.8)	
Health insurance status				
Insured	452	76.4 (70.6 82.2)	78.3 (73.1 83.5)	0.642
Uninsured		23.6 (17.8 29.4)	21.7 (16.5 26.9)	

Source: WOTRO-COHEiSION Ghana Project (2014); ^**^Pearson Chi-square test significant (*p* < 0.05); *Pearson Chi-square test significant (*p* < 0.1)

^a^Mean testing done with the paired *t*-test at 95 % confidence level

^b^Observations are the pooled responses of staff at baseline and follow-up

^c^Staff who performed clinical roles; ^d^staff who performed non-clinical roles

### Staff experiences and perception on the NHIS

**Fig. 2:**
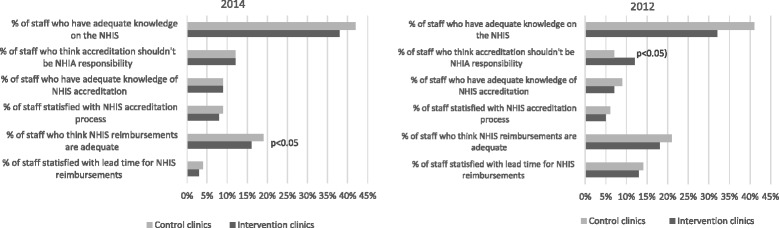
Health workers views on the NHIS in intervention and control clinics. Source: WOTRO-COHEiSION Ghana Project baseline and follow-up field data (2014); Legend: NHIS (National Health Insurance Scheme); NHIA (National Health Insurance Authority)

Experiences of health workers with some service components of the NHIS appeared to have worsened over time in both intervention and control facilities; the percentage of staff satisfied with timeliness of provider reimbursement decreased from approximately 14 % in 2012 to less than 10 % in 2014. Perceptions on the NHIS accreditation and information dissemination however improved marginally (see Fig. 2).

### Impact of interventions on staff perceptions of service quality

Difference-in-difference estimation results suggest the interventions impacted more positively on perceived availability and quality of drugs covered by the NHIS, contrary to anecdotal reports suggesting otherwise; average perception score in intervention clinics was 2.71 (SD = 0.20) compared to 2.51 (SD = 0.20) in controls (*p* < 0.05) (see Table 3). Perceived effect of the NHIS on increased workload on health staff and infrastructure appeared to have improved among respondents in intervention facilities (*p* < 0.1); likewise, client waiting and quality of time spent per client were perceived more positively by staff in intervention facilities than those in control facilities (see Table 3).

**Table 3:** Differences in health worker views on the NHIS in treatment and control facilities

		Baseline (2012)			Follow-up (2014)			Diff-in-Diff
		Control	Treated	Diff(BL)	Control	Treated	Diff(FU)	
Factor-analyzed variables^a^	^b^Obs	Mean(SE)	Mean(SE)	Mean(SE)	Mean(SE)	Mean(SE)	Mean(SE)	Mean(SE)
Perspectives on NHIS								
Feedback channels and stakeholder engagement	415	1.80 (0.19)	1.77(0.21)	−0.04(0.10)	1.70(0.20)	1.70(0.20)	0.00(0.10)	0.04(0.13)
Information provision, adequacy, accessibility	75	2.10(0.41)	1.81(0.44)	−0.24(0.18)	1.79(0.460	1.91(0.45)	0.11(0.22)	0.35(0.24)
Availability and quality of drugs covered by NHIS	429	2.64(0.21)	2.51(0.23)	−0.13(0.10)	2.51(0.20)	2.71(0.20)	0.20(0.09)**	0.33(0.14)**
Reimbursements and benefits package	200	1.55(0.22)	1.49(0.21)	−0.06(0.07)	1.55(0.22)	1.49(0.21)	−0.06(0.07)	0.00(0.00)
Trustworthiness and complaint handling	412	2.30(0.19)	2.25(0.22)	−0.05(0.09)	2.31(0.21)	2.40(0.21)	0.09(0.090)	0.14(0.11)
Overall perception score	40	2.37(0.55)	2.30(0.58)	−0.07(0.17)	2.37(0.58)	2.30(0.58)	−0.07(0.17)	0.00(0.00)
NHIS impact on quality care								
Workload and impact on health resources	421	1.82(0.21)	2.01(0.21)	0.19(0.08)**	1.67(0.23)	1.79(0.22)	0.12(0.07)*	−0.07(0.10)
Client waiting time and queuing system	425	0.95(0.11)	0.97(0.11)	0.02(0.05)	0.91(0.10)	0.97(0.10)	0.06(0.07)	0.05(0.10)
Quality of time spent per client	419	1.30(0.24)	1.39(0.28)	0.09(0.09)	1.25(0.25)	1.29(0.24)	0.05(0.06)	−0.05(0.12)
Overall perceived impact	419	1.53(0.13)	1.65(0.14)	0.13(0.06)**	1.42(0.13)	1.51(0.13)	0.09(0.05)*	−0.04(0.08)

Source: WOTRO-COHEiSION Ghana Project (2014); Diff.-in-diff estimates*p < 0.1; **p < 0.05. Note: Means and SE are bootstrapped and estimated by linear regression & all mean and SE values rounded up to the nearest decimal

Legend: *SE* Standard Error, *FU* Follow-up survey, *BL* Baseline survey

^a^Motivation factors have been factor-analyzed

^b^Observations are the pooled responses of staff at baseline and follow-up

Table 4 shows a model specification for the propensity score matching performed in Table 5. As shown in the propensity score matching, the SCE interventions seemed to enhance respondents’ perceptions on “drug availability and quality” (Average treatment effect on the treated (ATT) = 2.56); “trustworthiness/complaint handling” (ATT = 2.48) and “information provision on the NHIS” (ATT = 2.46). Moreover, the interventions apparently improved perspectives on effect of NHIS on “workload and health resources (ATT = 2.07) followed by “quality of time spent per client” (ATT = 1.38) and “client waiting time” (ATT = 1.18) (see Table 5).

**Table 4:** Model specification for propensity score matching using pooled 2014 & 2012 data (*n* = 468)

Variables	Variable definition	Intervention		Control		Difference in means
		*N* = 215 (46 %)		*N* = 253 (54 %)		
		Mean	SD	Mean	SD	
Treatment variable						
SCE/NSCE	1 if SCE clinic; 0 otherwise					
Outcome variables^a^						
Perception factor1	Perception factors (factor-analyzed)	1.92	0.67	1.94	0.61	0.02
Perception factor2		2.43	0.58	2.46	0.52	0.03
Perception factor3		2.55	0.70	2.53	0.65	−0.02
Perception factor4		1.74	0.53	1.79	0.49	0.05
Perception factor5		2.48	0.62	2.48	0.56	−0.00
Overall score		2.26	0.26	2.30	0.41	0.04
Perceived impact1		2.09	0.56	1.94	0.57	−0.15**
Perceived impact2		1.18	0.49	1.13	0.36	−0.05
Perceived impact3		1.38	0.59	1.31	0.49	−0.07
Overall score		1.73	0.39	1.62	0.38	−0.11**
Independent variables	(co-variates)					
Age	Staff age in years	37.4	13.1	37.6	13.1	−0.13
Gender	1 if male; 0 otherwise	0.40	0.49	0.30	0.46	0.09**
Education	1 if secondary education; 0 otherwise	0.28	0.45	0.21	0.40	0.07*
Profession	1 if non-clinical staff; 0 otherwise	0.26	0.44	0.24	0.44	0.02
Salary	1 if is > GHC 1,300; 0 otherwise	0.07	0.26	0.10	0.30	−0.03
Marital status	1 if married; 0 otherwise	0.44	0.50	0.45	0.50	−0.01
Religion	1 if Christian; 0 otherwise	0.97	0.18	0.96	0.20	0.01
Facility location	1 if GAR; 0 otherwise	0.51	0.50	0.52	0.50	0.01
Facility ownership	1 if private clinic; 0 otherwise	0.59	0.49	0.47	0.50	−0.12**
Insurance status	1 if insured; 0 otherwise	0.76	0.43	0.78	0.41	0.02

Source: WOTRO-COHEiSION Ghana Project (2014); Note: *SCE* Systematic community engagement intervention, *NSCE* No Community engagement intervention; SD (standard deviation); *p < 0.1; **p < 0.05; ^a^Staff perception variables factor-analyzed

**Table 5:** Effect of community engagement interventions on health worker perceptions (*n* = 468)

Matching algorithm	Outcome Indicators	^a^ATT (T-stat)	SE	Number of Intervention	Number of Control
Nearest Neighbor (NN)	Perception factor1	1.94(−0.26)**	0.080	194	221
	Perception factor2	2.46(−0.71)	0.165	36	39
	Perception factor3	2.56(−0.25)**	0.089	200	229
	Perception factor4	1.72(−1.37)*	0.099	98	102
	Perception factor5	2.48(−0.46)**	0.082	194	218
	Overall	2.24(0.13)	0.178	21	17
	Perceived impact1	2.07(2.60)**	0.074	198	223
	Perceived impact2	1.18(1.36)**	0.062	201	223
	Perceived impact3	1.38(1.44)**	0.067	201	224
	Overall	1.72(3.66)**	0.052	198	221

Source: WOTRO-COHEiSION Ghana Project (2014); *Psuedo-R2 (*p* < 1.0); **Pseudo-R2 (*p* < 0.05)

^a^ATT (Average treatment effect on the treated). The ATT values are the propensity score matching output and they depict the impact of the treatment (SCE interventions) on each of the staff motivation markers, high values imply higher treatment effect and *vice versa*

Legend: SE (Standard Error); Perception factor1 (Feedback channels and stakeholder engagement); Perception factor2 (Information provision, adequacy, accessibility); Perception factor3 (Availability and quality of drugs covered by NHIS); Perception factor4 (Reimbursements and benefits package); Perception factor5 (Trustworthiness and complaint handling); Overall perception (Overall score based on all five perception variables). Perceived impact1 (Workload and health resource); Perceived impact2 (Client waiting time and queuing system); Perceived impact3 (Quality of time spent per client); Overall perceived impact (Overall score based on all 3 perception variables on impact of NHIS on quality health service delivery)

## Discussion

Quality of services in NHIS-accredited health facilities and NHIS district offices, coupled with delayed reimbursement of service providers and administrative lapses remain critical challenges that have the potential of decreasing stakeholders’ trust and confidence in Ghana’s NHIS [10–14, 34, 35].

Bottom-up engagement of clients, healthcare providers and health insurance managers could be a possible strategy towards improving service quality at the health facility and district NHIS office levels. The results of this study suggest that SCE in service quality assessment could help enhance stakeholders’ perspectives on the NHIS and promote their goodwill, interest and support for NHIS activities. It was found that the average perception scores of respondents on the NHIS significantly improved after implementation of the SCE interventions (see Tables 3 and 5).

The interventions appeared to have enhanced perspectives on many components of the NHIS except delayed reimbursement of service providers, thus corroborating findings by Dalinjong and Laar [15] and Fusheini et al. [36] on this administrative challenge. Reports of provider claims being in arrears for several months [22] were confirmed in this study.

Even though NHIS reimbursements continue to be a key cost driver of the NHIA (escalating from US$ 3.4 in 2005 to nearly US$ 318 million in 2012 [1]), it appears the financial resources are inadequate and possibly constitute a cause of the delayed payment of service providers. The SCE had limited capacity to influence timely reimbursement of service providers which perhaps explains the apparent low influence on respondents’ perceptions.

The NHIA (regulatory body of the NHIS) established three zonal Claims Processing Centres (CPCs) with computerized claims vetting processes as part of efforts towards addressing the challenge of delayed reimbursements. Other strategies include routine reminders to accredited health facilities to submit claims on time and pick up payment cheques [1].

Proposals have also been made for review of the benefits package, and exemption list which are argued to be too generous and unsustainable [20]. Expansion of the NHIS revenue sources to include road tax, “sin” tax (i.e. tobacco and alcohol taxes) and petroleum revenue tax are being advocated to guarantee financial viability and early payment of service providers [10]. Comprehensive stakeholder consultation and policy dialogues could help explore the opportunities and potential challenges in implementing these proposals.

Frontline health workers’ perceptions on the adequacy of information dissemination by the NHIA improved marginally in both intervention and control facilities suggesting the need for intensified efforts in information dissemination strategies. The NHIA introduced a national call center in 2013 to address clients and service providers’ grievances on the NHIS. According to the NHIA, mass media campaigns to promote information accessibility to stakeholders have also been intensified [1]. Impact of these interventions on improved service quality however remain scientifically unproven.

The perceived impact of NHIS on quality health service delivery was found to have improved, particular among staff working in intervention health facilities (*p* < 0.05). Similar studies on impact of community engagement on quality care in Ghana [29] and health insurance in South Africa [22], Burkina Faso [24, 37], Rwanda [38] and Brazil [25] underscore the relevance of community engagement in health programmes including health insurance. Indeed, the African Union (AU) endorsed community engagement for health systems in Africa towards attaining sustainable and equitable health programmes [39].

These revelations demand increased political will to ensuring full engagement of communities and relevant stakeholders in health and insurance programmes. Perhaps, appraising performance of district and regional health administrations based on the level of community engagement in health provision could promote the concept. Likewise, evaluating performance of municipal and district assemblies (M&DAs) and members of parliament (MPs) based on the level of community engagement in health in their districts and constituencies could enhance political will and commitment.

The SCE interventions are potentially scalable and sustainable since they do not demand huge financial commitment. Approximately US$ 380.0 can conduct a round of SCE within the year. This modest financial commitment can easily be accommodated by monitoring budgets of the District Health Management Teams (DHMTs) and NHIS district offices [1].

Policy makers at the NHIA and Ministry of Health (MoH) levels could initiate discussions on possibly piloting and scaling up the SCE concept, preferably in the northern and middle belts of the country. The SCE concept could contribute towards empowering communities and creating a common platform for relevant stakeholders to deliberate on challenges and achievements of the NHIS and healthcare system at large.

National health policy direction on structured community engagement in health could help promote service provider accountability to clients and communities which would likely culminate in enhanced trust in the healthcare system and ultimately stimulate active participation in the NHIS and promote the pursuit for universal health coverage.

## Limitations

This study was conducted in clinics/health centres whose frontline workers might have significantly different perceptions and experiences of the NHIS from those working in bigger health facilities. Moreover, the study was conducted in two (2) out of ten (10) regions in the better endowed southern Ghana. Perhaps perspectives of health workers in the less endowed northern sector might be significantly different. Conclusions should therefore be interpreted with respect to validity for other regions and higher level healthcare facilities.

Moreover, given the time lag (2 years) between the baseline and follow-up surveys, it is possible some institutional and national level developments (beyond the control of this study) might have influenced the health staff perspectives on the NHIS besides the impact of the interventions. For instance, upgrading of some clinics/health centres to hospitals during follow-up could have affected, service delivery, human and material resource capacity and workload in these pertinent health facilities and hence impact on staff experiences and perceptions.

## Conclusions

Frontline health workers’ perceptions on the NHIS and its impact on quality health service delivery are predominantly positive especially in intervention health facilities. However, majority of staff expressed disappointment in delayed reimbursement of service providers implying the need for intensified efforts by NHIA and Government of Ghana (GoG) to ensure timely release of funds from the statutory National Health Insurance Fund (NHIF). Proposals for additional sources of revenue for the NHIS should also be discussed and possibly adopted to address this perennial challenge.

As demonstrated in this study, effective community engagement and stakeholder consultation is a viable strategy worth considering by policy makers to help promote stakeholder participation and support for the NHIS in Ghana. Accelerated political will and policy dialogues could help prioritize this proposal in the national agenda.

## Abbreviations

ATT, Average Treatment Effect on the Treated; AU, African Union; CPCs, Claims Processing Centres; DHMT, District Health Management Team; DiD, Difference-in-difference; ERC, Ethical Review Committee; GAR, Greater Accra region; GHS, Ghana Health Services; GoG, Government of Ghana; LE, Light Engagement; LMICs, Low and Middle Income Countries; M&DAs, Municipal and District Assemblies; MDGs, Millennium Development Goals; MoH, Ministry of Health; MPs, Members of Parliament; NHI, National Health Insurance; NHIA, National Health Insurance Authority; NHIF, National Health Insurance Fund; NHIS, National Health Insurance Scheme; OOP, Out of Pocket Payment; OPD, Out-patient Department; RCT, Randomized Control Trial; SCE, Systematic Community Engagement; SHI, Social Health Insurance; WR, Western region
